# Role of migration in the development of a first episode of psychosis

**DOI:** 10.1192/j.eurpsy.2022.1623

**Published:** 2022-09-01

**Authors:** R. Vaz, J. Martins, A. Costa, J. Brás, R. Sousa, E. Almeida, J. Abreu, D. Teixeira, A. Marques, N. Gil

**Affiliations:** Centro Hospitalar Tondela-Viseu , Departamento De Psiquiatria E Saúde Mental, Viseu, Portugal

**Keywords:** First Episode Psychosis, migration

## Abstract

**Introduction:**

Currently, there is scientific evidence supporting the relationship between socio-environmental factors and the onset of a first episode of psychosis (FEP). In this context, the phenomenon of migration, seen as a negative life experience, may become an important risk factor in developing a psychotic disorder (PD). In Europe, the impact of this phenomenon is growing and, therefore, it’s necessary to provide a proper answer to these individual’s mental health problems.

**Objectives:**

Identify which phases of this migration process are most important in the development of a FEP and what are the more significant socio-environmental factors in each phase.

**Methods:**

Bibliographic research in Pubmed database using the terms “Migration” and “First Episode Psychosis”.

**Results:**

Research confirms that migrants have a 2 to 3-fold increased risk of developing a PD. This risk will be even higher in the refugee population. Pre- and post-migration factors demonstrated to be more important than factors related with the migration process itself. In the pre-migration phase highlight factors like the lower parental social class and a previous trauma. In the post-migration phase highlight factors like discrimination, social disadvantage and a mismatch between expectations and reality.

**Conclusions:**

Literature is unanimous in considering migrant status as an independent risk factor for the development of FEP, possibly due to the outsider’s role in society. Thus, despite the growing interest in Biological Psychiatry, this work demonstrates that socio-environmental factors are very preponderant in the development of these disorders and because of that further investigation is still necessary.

**Disclosure:**

No significant relationships.

